# Cerebrovascular MRI in the mouse without an exogenous contrast agent

**DOI:** 10.1002/mrm.28129

**Published:** 2019-12-17

**Authors:** Jérémie P. Fouquet, Réjean Lebel, Lindsay S. Cahill, John G. Sled, Luc Tremblay, Martin Lepage

**Affiliations:** ^1^ Department of Nuclear Medicine and Radiobiology Faculty of Medicine and Health Sciences Université de Sherbrooke Sherbrooke QC Canada; ^2^ Mouse Imaging Centre The Hospital for Sick Children Toronto Ontario Canada; ^3^ Department of Medical Biophysics University of Toronto Toronto Ontario Canada

**Keywords:** $T_{2}^{*}$‐weighted MRI, anesthesia, cerebral angiography, mouse brain

## Abstract

**Purpose:**

To assess the effect of a variety of anesthetic regimes on $T_{2}^{*}$‐weighted MRI of the mouse brain and to determine the optimal regimes to perform $T_{2}^{*}$‐weighted MRI of the mouse cerebrovasculature without a contrast agent.

**Methods:**

Twenty mice were imaged with a 3D $T_{2}^{*}$‐weighted sequence under isoflurane, dexmedetomidine, or ketamine‐xylazine anesthesia with a fraction of inspired oxygen varied between 10% and 95% + 5% CO_2_. Some mice were also imaged after an injection of an iron oxide contrast agent as a positive control. For every regime, whole brain vessel conspicuity was visually assessed and the apparent vessel density in the cortex was quantified and compared.

**Results:**

The commonly used isoflurane anesthetic leads to poor vessel conspicuity for fraction of inspired oxygen higher or equal to 21%. Dexmedetomidine and ketamine‐xylazine enable the visualization of a significantly larger portion of the vasculature for the same breathing gas. Under isoflurane anesthesia, the fraction of inspired oxygen must be lowered to between 10% and 14% to obtain similar vessel conspicuity. Initial results on automatic segmentation of veins and arteries using the iron oxide positive control are also reported.

**Conclusion:**

$T_{2}^{*}$‐weighted MRI in combination with an appropriate anesthetic regime can be used to visualize the mouse cerebrovasculature without a contrast agent. The differences observed between regimes are most likely caused by blood‐oxygen level dependent effects, highlighting the important impact of the anesthetic regimes on cerebral blood oxygenation of the mouse brain at rest.

## 1 INTRODUCTION

The critical role of the vasculature in both the normal and pathological brain is increasingly drawn to our attention. In vivo imaging of the mouse cerebrovasculature has, therefore, the potential to reveal crucial information for a panoply of preclinical models.

MRI performed with $T_{2}^{*}$‐weighted or susceptibility‐weighted sequences has proven to be an efficient tool to study the human cerebrovasculature without contrast agent, e.g., with susceptibility weighted imaging (SWI) protocols.^1^ These methods rely on the blood oxygenation level‐dependent (BOLD) effect to reveal vessels containing sufficiently high levels of deoxyhemoglobin, typically veins.

When performing rodent imaging, the oxygen saturation of hemoglobin (SO_2_) is heavily affected by the anesthetic regime used. It was shown in rats that the convenient and widely used isoflurane (ISO) anesthetic increases cerebral SO_2_ compared with *α*_2_‐adrenoreceptors agonists anesthetics such as ketamine‐xylazine (KX) or dexmedetomidine (DEX), generating a loss of contrast between veins and cerebral tissues.^2^, ^3^, ^4^ However, this has not prevented the use of ISO anesthesia to study the rat cerebrovasculature without contrast agent with a $T_{2}^{*}$‐weighted sequence.^5^, ^6^

There is strong evidence that modulating the anesthetic regime can lead to a good vessel conspicuity in the mouse; this was reported in the first papers on BOLD by Ogawa and collaborators.^7^, ^8^ However, to the best of our knowledge, no previous work has been published using $T_{2}^{*}$‐weighted MRI ($T_{2}^{*}$ MRI) without contrast agent to study the mouse cerebrovasculature. $T_{2}^{*}$‐ or T2‐weighted imaging of the mouse cerebrovasculature is usually performed under ISO anesthesia with a contrast agent.^9^, ^10^, ^11^, ^12^ The contrast agent increases the complexity of the imaging experiment and can compromise vessel‐to‐tissue contrast in regions with increased vascular permeability.

In this study, we attempt to find an optimal anesthetic regime to image the vasculature of the mouse brain using $T_{2}^{*}$ MRI without contrast agent. We directly compare the cerebrovascular trees obtained under ISO, KX, and DEX and assess how modifying the breathing gases modulates image contrast and vessel conspicuity. These 3 anesthetics are already used extensively for mouse imaging studies because of practical aspects such as ease of use, the possibility of repeated use, and the quick recovery of the animals (especially for ISO and DEX).^13^, ^14^, ^15^, ^16^, ^17^ We also acquire $T_{2}^{*}$‐weighted images after an injection of an iron oxide contrast agent (Resovist) as a positive control and explore its capacity to differentiate veins and arteries in the cerebral cortex. For the first time, we present whole‐brain mouse vascular trees obtained with $T_{2}^{*}$‐weighted MRI without contrast agent showing a significant fraction of the vasculature.

The results presented are of practical relevance not only for researchers interested in maximizing vessel conspicuity to study the mouse brain vasculature, but also for those interested in minimizing vessel conspicuity to remove confound during the study of other phenomena involving $T_{2}^{*}$ MRI such as cerebral hemorrhage imaging, (targeted) particles of iron oxide imaging, and tracking of iron‐labeled cells. In addition, the results provide information on the effects of different anesthetics on the cerebral blood oxygenation of the mouse at rest.

## 2 METHODS

### 2.1 Anesthetic regimes

We tested a variety of anesthetic regimes. A single dosing was used for each anesthetic: for ISO, 1.5% in gas flowing at 1.5 L/min; for DEX (Precedex, Pfizer, New York, NY), initial bolus of 50 µg/kg and continuous infusion of 100 µg/kg/h started 8 min after initial bolus (injections in the caudal vein); for KX, mixture of ketamine (87 mg/kg) and xylazine (13 mg/kg) injected intraperitoneally. $T_{2}^{*}$ MRI was started 30 min after the anesthetic was administered for ISO and KX. For DEX anesthesia, mild ISO anesthesia was used for cannulation and was discontinued 2 min after the DEX bolus. $T_{2}^{*}$ MRI was then started 20 min after bolus injection. DEX anesthesia was reverted after imaging using a subcutaneous injection of 1 mg/kg of atipamezole (Revertor, Modern Veterinary Therapeutics, Miami, FL). Under all anesthetic regimes, mice were breathing freely in a nose cone through which a gas was administered at 1.5 L/min. We tested 3 different gases under DEX and KX anesthesia: carbogen (5% CO_2_ + 95% O_2_), 100% O_2_, and medical air (21% O_2_ + 79% N_2_). Under ISO anesthesia, we tested 100% O_2_, medical air, 18% O_2_, 14% O_2_, and 10% O_2_. The balance of the latter 3 gas mixes was N_2_. A scavenging system eliminated expiratory gases. The nose cone was carefully positioned on the animals to maintain residual expiratory gases at similar levels, although their level was not measured. Respiration rate was monitored, and mice were kept warm using a flow of air continuously adjusted at 30°C.

### 2.2 Animal groups

This study comprised a total of 86 imaging datasets. A total of *N* = 20 Balb/c mice of age 50 to 70 days were imaged with $T_{2}^{*}$ MRI under different anesthetic regimes. The mice were separated in 3 groups for which the anesthetic regimes are listed in Table 1.

**Table 1:** Anesthetic regimes under which the 3 animal groups of this study were imaged with the $T_{2}^{*}$ MRI sequence

Group 1 (*N* = 6 males)	Group 2 (*N* = 4 males and *N* = 4 females)	Group 3 (*N* = 6 males)
ISO/100% O_2_	ISO/medical air	ISO/medical air
KX/carbogen	DEX/carbogen	ISO/18% O_2_
KX/100% O_2_	DEX/100% O_2_	ISO/14% O_2_
KX/medical air	DEX/medical air	ISO/10% O_2_
ISO/100% O_2_ + Resovist		

For each group, imaging sessions were at least 1 week apart to ensure anesthetics were eliminated before the next session. The anesthetic regimes were tested in a random order, which was different for each mouse to randomize the effects of time and previous anesthesia on the results. For group 1, a last $T_{2}^{*}$ MRI acquisition under ISO/100% O_2_ was performed 2 min after the injection of a superparamagnetic particle of iron oxide (Resovist, Schering AG, Berlin, Germany; 5.6 mg of iron/kg through a caudal vein catheter). This method (referred to as ISO/Resovist) allows to visualize a large number of vessels in the mouse brain,^11^, ^12^ thus providing a positive control for vessel detection. The investigator was not blind to anesthetic conditions. All experiments were conducted in accordance with the recommendations of the Canadian Council on Animal Care and approved by the Institutional Animal Care and Use Committee (Comité Facultaire de Protection des Animaux de la Faculté de Médecine et des Sciences de la Santé; CFPA‐FMSS). Animals were kept in standard housing conditions with 14 h/10 h light/dark cycles, water and food ad libitum, and a maximum of 4 animals per cage. Animal experiments are reported in compliance with the ARRIVE (Animal Research: Reporting of In Vivo Experiments) guidelines.

### 2.3 $T_{2}^{*}$‐weighted MRI

$T_{2}^{*}$‐weighted MRI was performed on a 7T scanner (Varian Inc., Palo Alto, CA) using a dedicated mouse head‐coil (RAPID MR International, Columbus, OH). A slab selective 3D gradient echo sequence was used with repetition time = 50 ms, echo time = 25 ms, flip angle = 15°, acquisition bandwidth = 26.2 kHz, field of view = 20 × 15 × 10 mm^3^, voxel size = 78.1 × 78.1 × 104.2 µm^3^, 2 averages and flow compensation in all directions.

### 2.4 Image processing and analysis

#### 2.4.1 Whole brain vascular tree extraction

Every $T_{2}^{*}$ MRI data set was zero‐filled to obtain a reconstructed isotropic 39.1 µm resolution. Inhomogeneities in image magnitude (principally caused by receive head‐coil sensitivity) were corrected using N4ITK.^18^ Briefly, N4ITK fits a low frequency spatially varying field to the image and divides the image by this field, reducing variations of low spatial frequencies in the measured signal. Two methods were tested to extract a whole brain vascular tree from the $T_{2}^{*}$ MRI data: (1) histogram‐based thresholding of the Frangi index obtained from an anesthetic regime with high vessel conspicuity (e.g., KX/medical air) and (2) for each mouse, registering and comparing an image with high vessel conspicuity (e.g., KX/medical air) to an image with low vessel conspicuity (e.g., ISO/100% O_2_). For method 1, the Frangi filter^19^ was applied on 10 gaussian scales and the resulting index was binarized using as a threshold the maximum of the histogram plus 8 times the half width at half maximum. For method 2, $\Delta R_{2}^{*}$ maps were computed according to

$$\Delta R_{2}^{*}=\ln\left(\frac{S_{h}}{S_{l}}\right)/\text{TE}$$

with *S_h_* and *S_l_* the MRI magnitude signal from the anesthetic regime with high and low vessel conspicuity, respectively, and TE the echo time.

#### 2.4.2 Differences between anesthetic regimes

**Figure 1:**
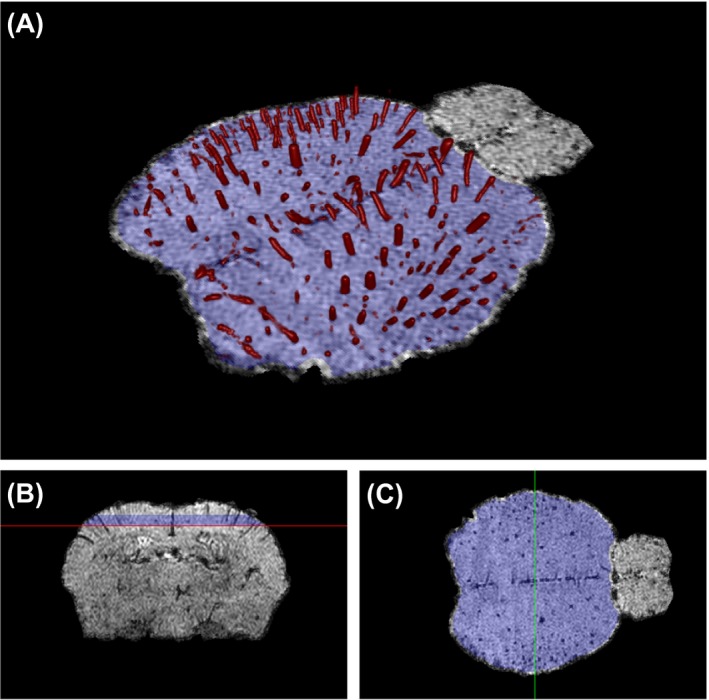
Illustration of the ROI defined in the cortex for AVD computation and discrimination between veins and arteries. The vessels segmented in the ROI (blue) for a specific representative scan are depicted in panel A (vessels are 3D rendered in red). The ROI overlaid on magnitude image is also shown in the axial (B) and coronal (C) planes

An average brain was created by registering with affine transformations 3 data sets obtained under KX/medical air anesthesia using ANTs software.^20^ A region of interest (ROI) was manually drawn on the cerebral cortex of the average brain for further analysis (Figure 1). The average brain and the ROI were registered to every data set using an affine transform. For every data set, the apparent vascular density (AVD) was computed in the ROI according to:

$$\text{AVD}=V_{\mathrm{vasc}}/V_{\mathrm{ROI}},$$

where *V_ROI_* is the volume of the registered ROI, and *V_vasc_* is the volume occupied by the extracted vasculature in the ROI. For comparison between anesthetic regimes, we use the vasculature tree obtained by thresholding the Frangi index (method 1 of the previous subsection). The statistical significance of the AVD difference between anesthetic regimes was tested within each group by 1‐way analysis of variance with a Tukey correction for multiple comparisons. For group 2, the effect of sex was also tested using a 2‐way analysis of variance with a Sidak correction for multiple comparisons. The differences were assumed to be significant when *P*‐values < 0.05. Statistical analyses were performed with Graphpad Prism (version 7.03, Graphpad Software Inc, La Jolla, CA).

#### 2.4.3 Vessel size analysis

**Figure 2:**
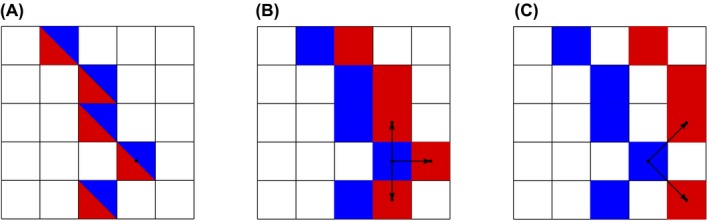
Various possible situations occurring when matching a point of a low AVD vessel skeleton (blue) to a high AVD vessel skeleton (red). A, The point from low AVD skeleton is matched to a unique point in the high AVD skeleton. B,C, the low AVD point is equally distant from 3 and 2 points from the high AVD skeleton, respectively. For further analysis, we match to the point from the lower AVD skeleton the average of the characteristics of the matching points in the higher AVD skeleton (for instance, the average vessel size of the multiple matched points in B and C)

The diameter of the vessels segmented by thresholding the Frangi index was estimated using an adapted version of the local thickness algorithm available in FIJI.^21^, ^22^ The vessel masks were skeletonized using a Matlab implementation of the algorithm proposed by Lee and collaborators.^23^, ^24^ For each mouse and for every pair of anesthetic regimes, we compared corresponding matching points in the skeleton of the vasculature. To find matching points, we first registered the data sets of individual mice using ANTs and a rigid transform. Then, for every pair of regimes, we attempted to match all the skeleton points of the lower AVD regime to that of the higher AVD regime based on the distance between points, with the condition that points were within 117 µm (3 voxels). When points in the lower vascular density skeleton could be matched to more than 1 point in the higher vascular density skeleton, the average of the vessel size of matched points in the higher AVD skeleton was used for further analysis (see Figure 2). Vessel size analysis was performed on the whole brain. For every pair of regimes, we also analyzed the new vessels detected in the higher AVD regime. To be considered as a new vessel, an independent component of the higher AVD skeleton needed to include more than 90% of newly detected points.

#### 2.4.4 Discrimination between veins and arteries

The proposed differentiation between veins and arteries uses an anesthetic regime that presumably reveals primarily veins, and the ISO/Resovist regime which reveals both veins and arteries. For the purposes of this study, we use the KX/medical air regime to reveal primarily veins. This choice relies on the hypothesis that arterial SO_2_ remains high under KX/medical air (see the Discussion section for further comments on this hypothesis). Therefore, the points of the ISO/Resovist skeleton that were matched to points in the KX/medical air skeleton (as described in the previous subsection) were assumed to be part of the venous system. On the contrary, points of the ISO/Resovist skeleton that could not be matched to points in the KX/medical air skeleton were considered to be part of the arterial system. The diameter of the venous and arterial vessels was computed by multiplying the corresponding skeleton by the vascular local thickness previously obtained. Discrimination between veins and arteries was only tested in the cortex ROI.

## 3 RESULTS

### 3.1 Effects of anesthetic regimes on $T_{2}^{*}$ MRI

**Figure 3:**
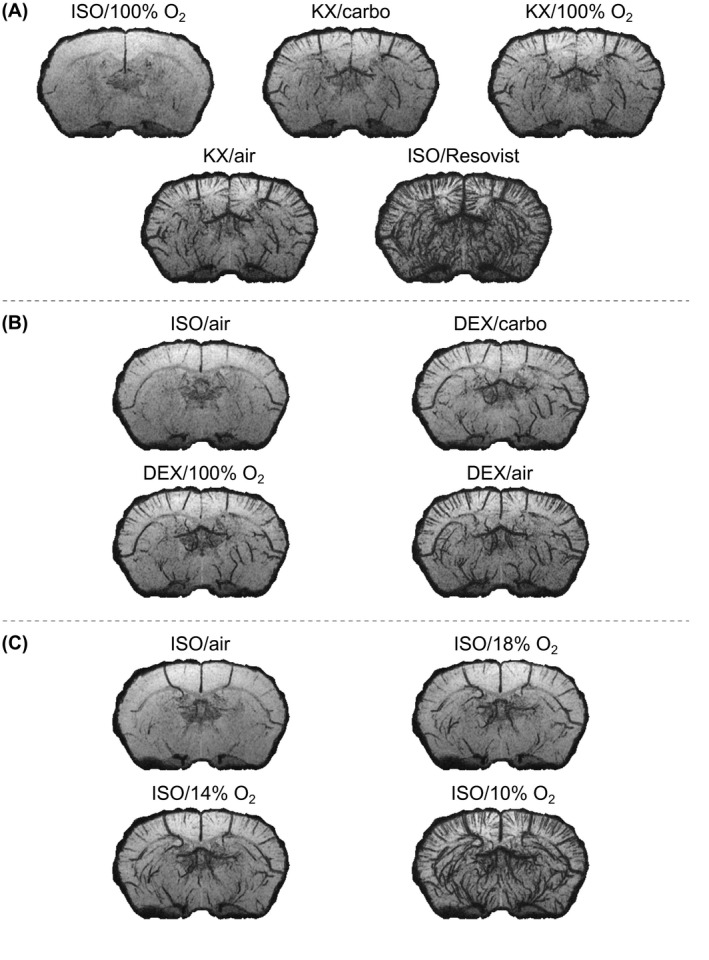
Minimum intensity projection over 1.21 mm of the magnitude images obtained under all regimes tested for a representative mouse in groups 1 (A), 2 (B), and 3 (C). The conspicuity of vessels depends strongly on the anesthetic regime. Air, medical air; carbo, carbogen

**Figure 4:**
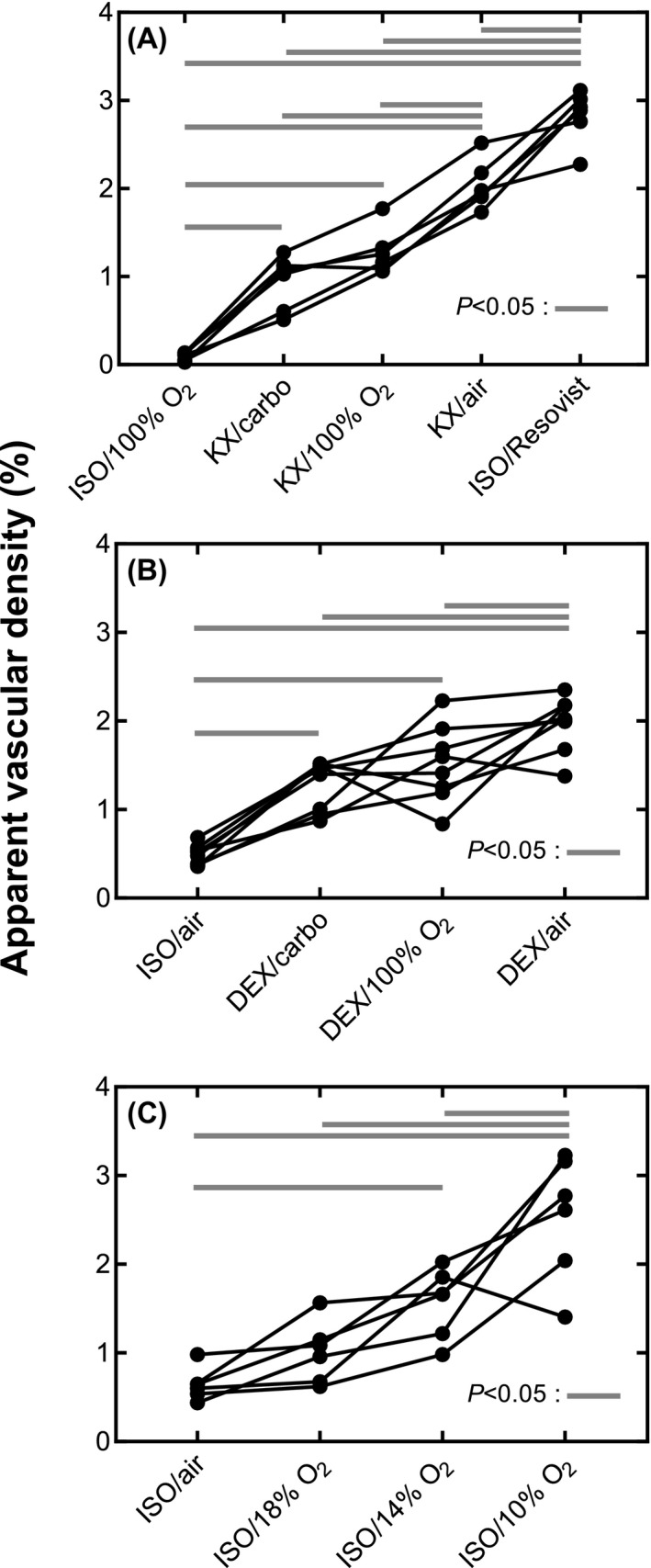
AVD computed in the cortex ROI for groups 1 (A), 2 (B), and 3 (C). Solid black lines represent individual animals. A solid gray horizontal line indicates a significant difference. The AVD measured from the mouse brain clearly depends on the anesthetic regime. Air, medical air; carbo, carbogen

The contrast in $T_{2}^{*}$ MRI images of the mouse brain is directly linked to the anesthetic regime (Figure 3). A striking feature is the large variation of the conspicuity of vessel‐shaped structures. Whereas DEX and KX anesthesia produce a similar apparent cortical vascular density, ISO anesthesia reveals a significantly lower AVD for an equivalent breathing gas (Figure 4). We investigated whether vessel conspicuity was also modulated by the breathing gas. For KX and DEX, medical air generates significantly higher AVD than carbogen and O_2_, while the latter 2 yield AVDs that are not significantly different. For ISO, using 100% O_2_ as a carrier leads to the detection of very few vessels (Figures 2 and 3). Reducing the O_2_ concentration increases the number of vessels detected: ISO/10% O_2_ yields significantly higher AVD than all other regimes, while ISO/14% O_2_ yields significantly higher AVD than ISO/medical air. Statistical tests showed no effect of the sex for the group where the 2 sexes were present (group 2; effect size of 0.4% and *P* = 0.55).

### 3.2 Whole brain vascular tree extraction

**Figure 5:**
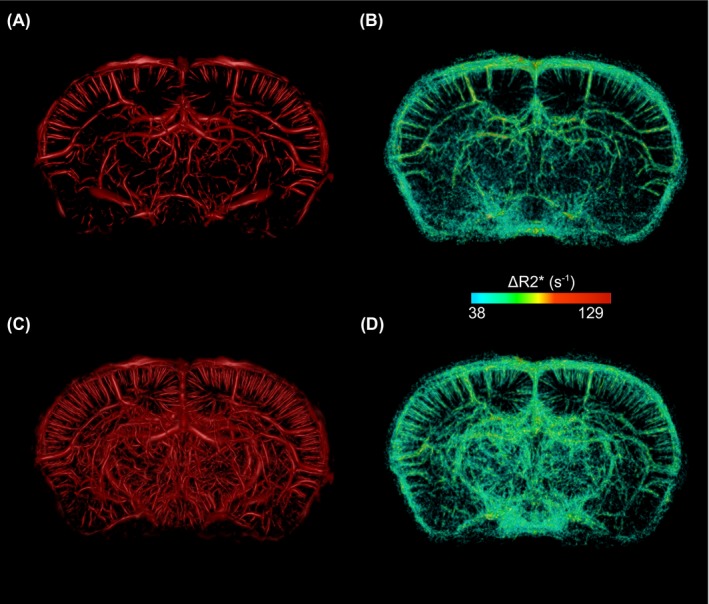
3D rendering of a 3.5‐mm‐thick section of the vascular trees extracted from KX/medical air data set (A,B) and from ISO/Resovist data set (C,D). The first column (A,C) shows the Frangi index. The second column shows $\Delta R_{2}^{*}$ obtained by comparison with the ISO/100% O_2_ regime. $\Delta R_{2}^{*}$ = 38 s^−1^ was empirically found to differentiate vascular structures from other brain tissues

Figure 5 shows vascular trees obtained from an anesthetic regime with high AVD (KX/medical air) by the 2 proposed methods (Frangi index and $\Delta R_{2}^{*}$ computation by comparison with ISO/100% O_2_). Both methods for vascular tree extraction yield similar vascular structures within the brain. Figure 5 also shows the vascular tree obtained with Resovist as a positive control. As reflected by the AVD within the cortex ROI (Figure 4), more vessels are present in the vascular tree obtained with Resovist. The $\Delta R_{2}^{*}$ computation reveals more structures at the surface of the brain than the Frangi index method. A $\Delta R_{2}^{*}$ value of 38 s^‐1^ was empirically found to be a good threshold to separate vascular structures from other brain tissues.

### 3.3 Discrimination between veins and arteries

**Figure 6:**
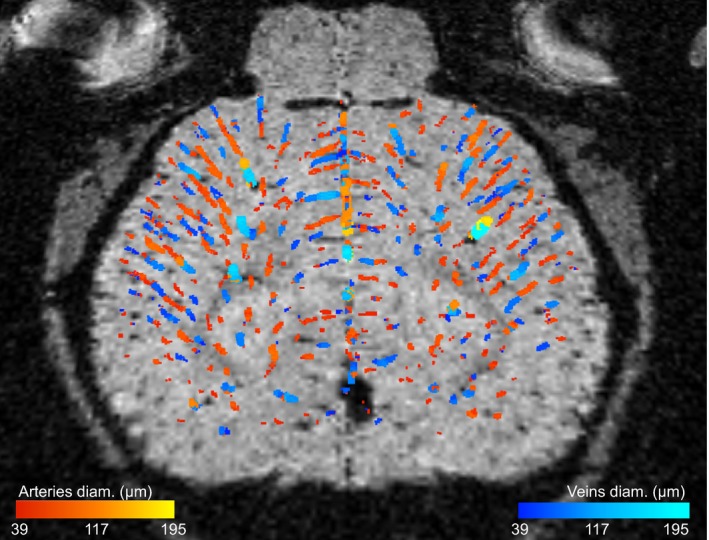
Veins and arteries segmented in the cerebral cortex region of interest using the automatic method proposed. The vessels are color coded for their diameter. Some veins appear to transform into arteries at their endpoint, which most likely reflects the threshold for vessel detectability

Figure 6 shows an example of the veins and arteries obtained in the cerebral cortex ROI of a mouse using the proposed automatic method. The spatial distribution of veins and arteries is relatively uniform across the ROI. Some veins appear to transform into arteries at their endpoint, which most likely reflects the threshold for vessel detectability.

### 3.4 Apparent vessel diameter comparison

**Figure 7:**
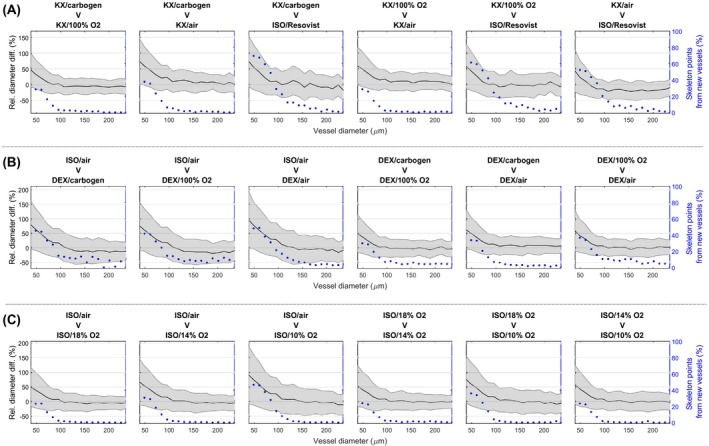
Relative changes in apparent diameter (left y axis; solid black line with shaded error bars) and percentage of skeleton points belonging to new vessels (right y axis; blue points) for every pair of regimes in group 1 (A; first row), group 2 (B; second row), and group 3 (C; third row). For each pair, the regime with lower AVD is indicated first over the graph. The changes in apparent vessel diameter (left y axis) are plotted as a function of the apparent diameter for the regime with lower AVD. The percentage of the skeleton belonging to new vessels (right y axis) is plotted as a function of the apparent vessel diameter for the regime with higher AVD. Values from the whole brain of all animals were pooled to create the graphs. Overall, left y axis shows that vessels appear larger in the regime detecting more vessels. Right y axis shows that it is principally small vessels in the regime detecting more vessels that are unmatched. For group 1, ISO/100% O_2_ is not presented because there were not enough vessels detected. Air, medical air; carbo, carbogen; Reso, Resovist; vasc., vascular

Figure 7 shows how the apparent vessel diameters differ between anesthetic regimes within the whole brain. For each pair of regimes, the relative difference in apparent vessel diameter is plotted as a function of the diameter in the lower AVD regime. For group 1, the ISO/100% O_2_ regime was not included in the comparison because not enough vessels were detected. For most of the regime pairs, the vessels diameter seems overestimated for smaller vessels (≤60 µm). Figure 7 also shows the percentage of points belonging to new vessels in the skeleton of the regimes with higher AVD. In general, the percentage of points belonging to new vessels is larger for smaller diameters. It is also larger for regime pairs with a more important AVD difference. For instance, the KX/carbogen V ISO/Resovist pair presents the highest percentages of points belonging to new vessels.

## 4 DISCUSSION

$T_{2}^{*}$ MRI techniques without contrast agent were proven useful to detect brain vasculature in the rat^5^, ^6^ and in humans^1^ but, to the best of our knowledge, applications in the mouse brain have not been reported. Our results directly show that it is possible to reveal detailed vascular architecture in the mouse brain using $T_{2}^{*}$ MRI without contrast agent (Figures 3, 4, 5).

KX and DEX produce a mouse vascular tree with similar AVD in the cortex ROI for equivalent breathing gas (Figure 4). For these anesthetics, even carbogen‐breathing allows for the visualization of vessels. To obtain similar vascular density under ISO anesthesia, fraction of inspired O_2_ must be lowered under 21%. A fraction of inspired O_2_ between 10% and 14% allows for the visualization of an AVD comparable to that obtained with DEX/medical air and KX/medical air. As expected, the ISO/Resovist regime generates the highest AVD.

The striking changes observed are most likely due to differences in cerebral SO_2_ between anesthetic regimes, deoxyhemoglobin generating most of the contrast seen in Figure 3. Similar contrast differences in $T_{2}^{*}$ MRI of the rat under different anesthetic regimes have been observed and attributed to SO_2_ changes.^3^, ^4^ Indeed, xylazine and DEX are both *α*_2_‐adrenergic receptors agonists that were shown to cause a significant reduction in rodents cerebral blood flow compared with ISO.^2^, ^13^, ^25^, ^26^ This is putatively caused by their cerebral vasoconstrictive^27^, ^28^ and significant bradycardic effects.^29^, ^30^ In contrast, ISO is known to have dose‐dependent vasodilatory^31^ and light bradycardic effects.^2^, ^29^, ^32^ It was also shown in dogs that DEX with low‐ISO anesthesia increases cerebral metabolic rate of oxygen compared with ISO‐only anesthesia.^33^ Similarly, increasing the ISO dose decreases the cerebral metabolic rate of glucose in rats.^26^ Taken together, these observations support the notion that SO_2_ in cerebral blood could be lower under DEX and KX than under ISO, especially on the venous side. This interpretation is further supported by the previous observation that, in rat, cerebral partial pressure of oxygen (pO_2_) was lower under KX anesthesia than under ISO anesthesia.^2^, ^34^, ^35^

Our results show that the widely used ISO anesthesia generates poor vessel conspicuity in the mouse when supplied in 100% O_2_ or medical air (Figures 2 and 3). In contrast, vessels are clearly detected under ISO anesthesia with similar gas conditions in rats.^5^, ^6^ These observations are consistent with results indicating that ISO anesthesia at a dose of 1.1‐fold the minimal alveolar concentration (mice: 1.8‐2.0%; rats: 2‐2.3%) exhausts the cerebrovascular reserve capacity in mice more effectively than in rats.^13^ This reduction of the cerebrovascular reserve capacity is attributed to cerebral vasodilation occurring in the resting state, which leads to an increase of the basal cerebral blood flow and possibly of the basal venous blood oxygenation. The smaller diameter of the mouse vessels can also be partly responsible for the lower number of vessels detected in this species.

Within the limits of our methodology, the interindividual variations appear lower with KX than with DEX or ISO/14% O_2_ and ISO/10% O_2_. However, ISO can be administered more easily and allows for a quick recovery, and DEX has the advantage of being easy to revert using atipamezole. The effects of KX will subside over time because it is injected intraperitoneally before imaging. This can limit the scanning time and introduce variability in the physiological parameters of the animal (including blood oxygenation) during the scan. The choice of the most appropriate anesthetic for an imaging study of the cerebrovasculature must include study‐specific information, such as the anesthetic administration method, the region(s) of interest in the brain, and the accessibility to anesthetics or breathing gases.

We tested the Frangi index and the $\Delta R_{2}^{*}$ methods to obtain a cerebrovascular tree from $T_{2}^{*}$ MRI data sets; both yielded similar vascular structures (Figure 5). The Frangi index method requires a single data set, but small vessels at the surface of the brain are not efficiently identified due to the lack of MR signal from the skull. Vessel‐to‐tissue contrast could potentially be further improved by performing the extra postprocessing steps of SWI before application of vessel extraction algorithms. However, SWI postprocessing contains a set of tunable parameters that would need to be optimized with respect to future end‐point applications as they may introduce unwanted variability to our method.^1^ The second method was based on the calculation of $\Delta R_{2}^{*}$, which requires 2 data sets. As expected, this method is superior for the detection of vessels at the surface of the brain.^5^ It also generates a quantitative measurement of $\Delta R_{2}^{*}$ which can characterize microvasculature.^36^ We note that the absolute $\Delta R_{2}^{*}$ values reported here are to be considered with caution because the scan under ISO/100% O_2_ and the scans under KX/medical air or ISO/Resovist were not acquired during the same imaging session. Nonuniformity between spatial distributions of image intensity may result in inaccurate $\Delta R_{2}^{*}$ values.

We explored the possibility of automatically differentiating veins and arteries by comparing images acquired with and without contrast agent. Our method, conceptually similar to a manual method proposed for the cat brain,^37^ produces a map of veins and arteries along with their diameter (Figure 6). Such maps could be useful to explore the role of the vasculature in various pathologies or to better understand the origin of the BOLD signal in fMRI.^38^, ^39^, ^40^, ^41^ The underlying assumptions are that (1) the vessels visible under KX/medical air were principally veins with contrast generated by deoxyhemoglobin, (2) the vessels visible under ISO/Resovist were both veins and arteries with contrast generated by Resovist, and (3) the veins visible under KX/medical air regime corresponded to veins detected under ISO/Resovist.

Assumption (1) is equivalent to the one used to claim that SWI of the human brain primarily reveals veins. It supposes that arterial SO_2_ remains high under the anesthetic regime chosen to reveal veins. Whether it is respected in the mouse under anesthetic regimes with high AVD such as KX/medical air is an interesting open question. Based on the intracortical location of newly detected vessels, it was suggested that arterial SO_2_ decreases sufficiently to allow detection with $T_{2}^{*}$ MRI in the rat brain under regimes similar to ISO/medical air.^6^ A control experiment differentiating veins and arteries would allow to directly answer this question in the mouse. This would help to further validate the automatic method presented here to separate veins and arteries. Also, it is apparent from the map presented in Figure 6 that assumption (3) does not hold perfectly for all vessels. For instance, end‐points of vessels identified as veins become arteries, which is not realistic. This instead suggests that ISO/Resovist revealed end‐points of veins that were undetectable under KX/medical air. The contrast of small veins can drop just under the detection threshold under KX/medical air. When Resovist is injected, the larger contrast enables detection of smaller portions of veins.

The apparent diameter of the detected vessels is a combination of the real vessel diameter, the magnetic susceptibility of blood, the angle between the vessel and the main magnetic field, the acquisition parameters, and the reconstruction parameters. In the tested regimes, the susceptibility of blood is affected by deoxyhemoglobin and/or by Resovist. We ran simulations with a 2‐compartments model,^42^ our acquisition parameters, and a conservative venous SO_2_ of 0.7^43^ to estimate the diameter of the smallest detectable vessels in the anesthetic regimes with low SO_2_ such as KX/medical air, DEX/medical air, and ISO/10% O_2_. Based on the acquisition voxel volume and a detectability threshold of the parenchyma signal minus 3 times the standard deviation of the noise, we obtained a minimum detectable diameter of 14 µm for a vessel perpendicular to B_0_ and of 51 µm for a parallel vessel. Zero‐filling is known to increase spatial resolution and mitigate partial volume effects.^44^, ^45^, ^46^ The theoretical relationship giving the smallest detectable diameter while considering zero‐filling and the Frangi filter is beyond the scope of this study.

Comparing the apparent vessel diameters in different regimes clarifies the origin of the AVD differences. For instance, the apparent diameters detected in the ISO/Resovist regime were comparable to those detected in the KX/medical air regime (Figure 7A). This suggests that the susceptibility of blood is comparable in those 2 cases where the concentration of Resovist coincidentally produced a similar susceptibility than the relative concentration of deoxyhemoglobin in the vessels. The higher AVD under ISO/Resovist, therefore, results from an increase in the total length of the vasculature detected, which was assumed to be arteries. The fact that the percentage of points belonging to newly detected vessels is higher for smaller diameters for all compared pairs (Figure 7) is attributed to 2 main causes: (1) false positive vessels due to noise are more likely to be of small diameter and (2) the small vessels detected under a higher AVD regime are often just under the detectability threshold under the lower AVD regime.

Our algorithm matching vessel skeleton points is useful in automatically separating veins and arteries and provides information on how apparent vessel diameters compare between 2 anesthetic regimes. It included an approach based on the Euclidian distance between skeleton points after rigid registration of brain images under different regimes. This approach could be improved by considering whole vessel segments during matching instead of single points, and by adding more conditions for vessel matching, e.g., proximity to an expected diameter difference.

It would have been relevant to compare the results presented in this study with independent measurements of blood gases and blood oxygenation. However, blood sampling (the most common and reliable method of measurement) is difficult to perform as part of survival studies in the mouse. Terminal blood sampling would have prevented repeated MRI scans in the same animals with the detrimental consequences of introducing more important inter‐individual differences and of increasing the total number of animals required in the study. The use of a pulse oximeter could have provided the arterial SO_2_, although its accuracy is limited in mice.^16^ Similarly, it would have been relevant to measure the concentration of inspired and expired breathing gases, but these measures are not performed easily in a noninvasive way in the mouse.^16^

## 5 CONCLUSIONS

We demonstrated that it is possible to image a significant fraction of the mouse cerebrovasculature using $T_{2}^{*}$ MRI without contrast agent by choosing an appropriate anesthetic regime. As opposed to results obtained in the rat, the widely used ISO anesthetic generates a poor vessel conspicuity when administered with 100% O_2_ or medical air. To improve vessel visualization, one can lower the fraction of inspired oxygen below 14% or use KX or DEX as an anesthetic. This is most likely due to a decrease in cerebral blood oxygenation under KX and DEX anesthesia, which is in line with results available for the rat^3^ and with the known physiological effects of the 3 anesthetics. We also presented groundwork for automatically segmenting veins and arteries by combining acquisitions with and without a contrast agent. It is expected that our methodology can provide vasculature maps that can be useful to study the role of vasculature in the normal or pathological mouse brain.
